# HLX and SLC25A20: Immunologic regulators bridging ankylosing spondylitis and uveitis via multi-omics integration and machine learning

**DOI:** 10.1371/journal.pone.0332049

**Published:** 2025-09-10

**Authors:** Weiming Cai, Huayan Chen, Dong Huang, Xiaoli Ma, Dongbo Jiang, Tangming Guan

**Affiliations:** 1 Department of Pharmacy, Affiliated Hospital of Guangdong Medical University, Zhanjiang, Guangdong Province, China; 2 Laboratory of Clinical Pharmacy, Affiliated Hospital of Guangdong Medical University, Zhanjiang, Guangdong Province, China; The University of Texas Health Science Center at Houston / McGovern Medical School, UNITED STATES OF AMERICA

## Abstract

**Background:**

Ankylosing spondylitis (AS), a chronic inflammatory disorder affecting axial joints, is frequently complicated by uveitis. However, the molecular mechanisms linking AS to secondary uveitis remain poorly understood.

**Methods:**

We integrated transcriptomic datasets from AS (GSE73754) and uveitis (GSE194060) cohorts to identify shared molecular pathways. Differential expression analysis, weighted gene co-expression network analysis (WGCNA), and machine learning (LASSO, SVM-RFE, random forest) were combined to prioritize biomarkers. Molecular docking was performed to evaluate drug-target interactions using Vina scores (≤−7 kcal/mol threshold).

**Results:**

Cross-disease analysis revealed 697 overlapping dysregulated genes (481 upregulated, 216 downregulated), enriched in GTPase signaling and immune pathways. WGCNA identified disease-specific co-expression modules (AS: brown/tan modules, *r* = 0.39/0.35; uveitis: brown module, *r* = 0.49). Machine learning nominated HLX and SLC25A20 as core biomarkers, demonstrating robust diagnostic accuracy in discovery (AS AUC: 0.688/0.700; uveitis AUC: 0.867/0.838) and validation cohorts (AS AUC: 0.653/0.667; uveitis AUC: 0.662/0.736). Immune profiling linked HLX to neutrophil infiltration (*r* = 0.55, *p* < 0.01) and SLC25A20 to T helper cell regulation (*r* = 0.36, p < 0.01). Molecular docking identified high-affinity ligands for SLC25A20, including amiodarone (−8.0 kcal/mol) and estradiol (−7.7 kcal/mol), with folic acid showing dual binding potential (HLX: −7.5 kcal/mol; SLC25A20: −8.2 kcal/mol).

**Conclusion:**

HLX and SLC25A20 emerge as immunologic regulators bridging AS and uveitis pathogenesis. These findings provide actionable targets for precision diagnostics and therapeutic development in AS-associated uveitis.

## Introduction

Ankylosing spondylitis (AS) is a chronic inflammatory disorder that primarily affects the axial joints and can lead to significant functional impairment [1,2]. One of the notable extra-articular manifestations of AS is uveitis, an inflammatory condition affecting the uveal tract of the eye. Approximately 30–40% of AS patients experience episodes of uveitis, often preceding the onset of axial symptoms and associated with a higher degree of systemic inflammation [3,4]. Despite the strong correlation between AS and HLA-B27 positivity, the specificity and sensitivity of HLA-B27 as a biomarker for diagnosing uveitis in AS patients remain suboptimal [5–7].

The IL-23/IL-17 inflammatory axis has been implicated in the pathogenesis of both conditions [8–10]; however, the underlying molecular mechanisms linking AS and uveitis are not fully understood. Research has shown that T cells and natural killer (NK) cells play critical roles in the development of AS [11–13], while oxidative stress and T cell receptor signaling dysregulation are closely associated with uveitis [11,14–16]. Nonetheless, most studies thus far have been conducted within the confines of individual disease frameworks, neglecting the exploration of shared pathways and common biomarkers that could elucidate the co-morbid nature of these disorders. Recent advances in high-throughput genomic technologies and bioinformatics have opened new avenues for understanding complex disease interactions. Machine learning (ML) techniques, in particular, have demonstrated an ability to integrate and analyze multi-omics datasets, revealing intricate gene-gene interactions and regulatory networks. However, the application of ML in the study of co-morbid conditions such as AS and uveitis remains largely unexplored.

**Fig 1:**
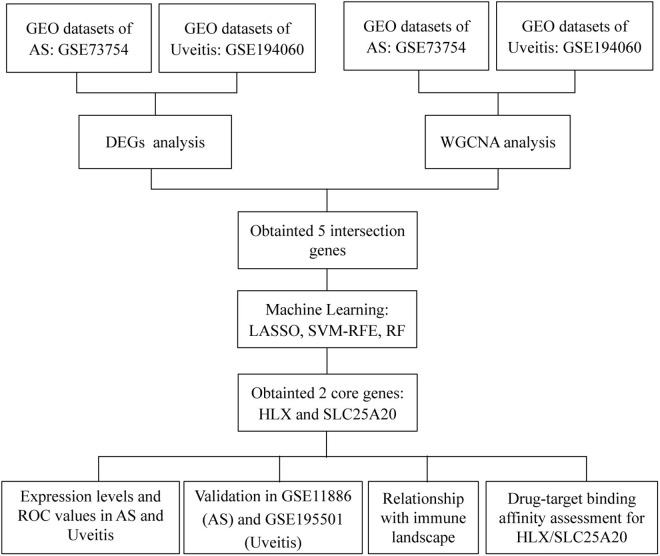
Flowchart of the study design. GEO: Gene Expression Omnibus; AS: ankylosing spondylitis; DEGs: differentially expressed genes; WGCNA: weighted gene coexpression network analysis; SVM-RFE: support vector machine-recursive feature elimination; RF: random forest.

In this study, we aim to systematically analyze the peripheral blood transcriptomic data from AS and uveitis patients to identify shared biomarkers and pathways. By employing differential expression analysis, weighted gene co-expression network analysis (WGCNA), and several machine learning algorithms including least absolute shrinkage and selection operator (LASSO), support vector machine recursive feature elimination (SVM-RFE), and random forest (RF), we seek to uncover disease-specific modules and potential biomarkers that link AS and uveitis. Furthermore, we will validate the diagnostic potential of these biomarkers and investigate their association with immune cell infiltration (Fig 1). Overall, our study offers worthy insight into the interconnected molecular pathways of AS and uveitis, and emphasizes the potential of HLX and SLC25A20 as diagnostic markers and targeted treatment approaches for patients suffering from these circumstances.

## Results

### Identification of cross-disease differentially expressed genes (DEGs)

**Fig 2:**
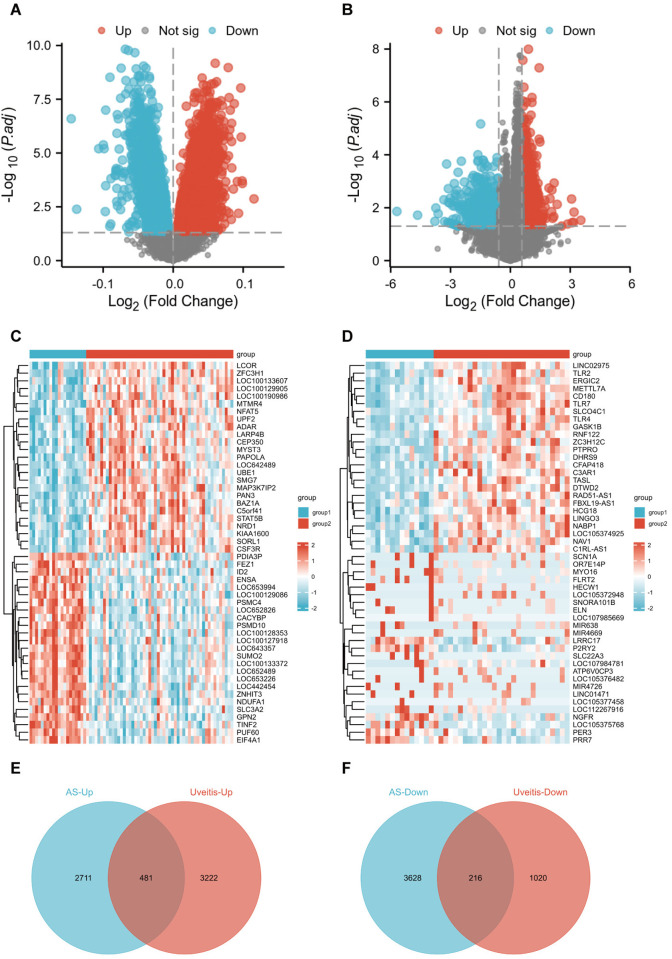
Identification of cross-disease DEGs. **(A, B)** Volcano plots illustrating DEG distribution in AS (GSE73754) and uveitis (GSE194060). **(C, D)** Heatmap visualization of DEGs in the AS cohort (GSE73754) and uveitis cohort (GSE194060). **(E, F)** Venn diagrams showing shared upregulated (481 genes) and downregulated (216 genes) DEGs between AS and uveitis.

To systematically identify shared transcriptional alterations in ankylosing spondylitis (AS) and uveitis, we performed differential expression analysis on peripheral blood transcriptomes using a significance threshold of *p <* 0.05 (no fold-change cutoff to maximize candidate genes). In the AS cohort (GSE73754), we detected 7,036 DEGs, comprising 3,192 upregulated and 3,884 downregulated genes (Fig 2A, 2C). The uveitis dataset (GSE194060) revealed 4,939 DEGs, with 3,703 upregulated and 1,236 downregulated transcripts (Fig 2B, 2D). A subsequent intersection of disease-specific DEGs yielded 481 shared upregulated genes and 216 shared downregulated genes between AS and uveitis cohorts (Fig 2E, 2F).

### Functional enrichment analysis of DEGs

**Fig 3:**
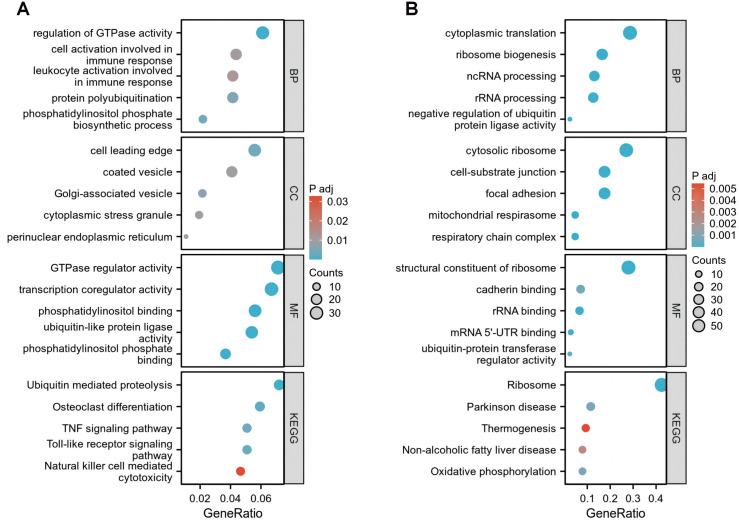
Functional enrichment analysis of DEGs. **(A)** GO and KEGG pathway enrichment of 481 shared upregulated DEGs. **(B)** GO and KEGG pathway enrichment of 216 shared downregulated DEGs.

We performed Gene Ontology (GO) and Kyoto Encyclopedia of Genes and Genomes (KEGG) enrichment analyses on the identified DEGs. The results demonstrated that the 481 shared upregulated DEGs were predominantly enriched in biological processes including regulation of GTPase activity, protein polyubiquitination, and cell activation involved in immune response. These genes were further mapped to key pathways such as ubiquitin-mediated proteolysis, osteoclast differentiation, and Toll-like receptor signaling pathway (Fig 3A). Similarly, the 216 downregulated DEGs were significantly associated with processes such as cytoplasmic translation, ribosome biogenesis, and rRNA and ncRNA processing. Pathway analysis revealed their involvement in ribosome assembly and oxidative phosphorylation (Fig 3B).

### WGCNA and key kodule identification in AS and uveitis

**Fig 4:**
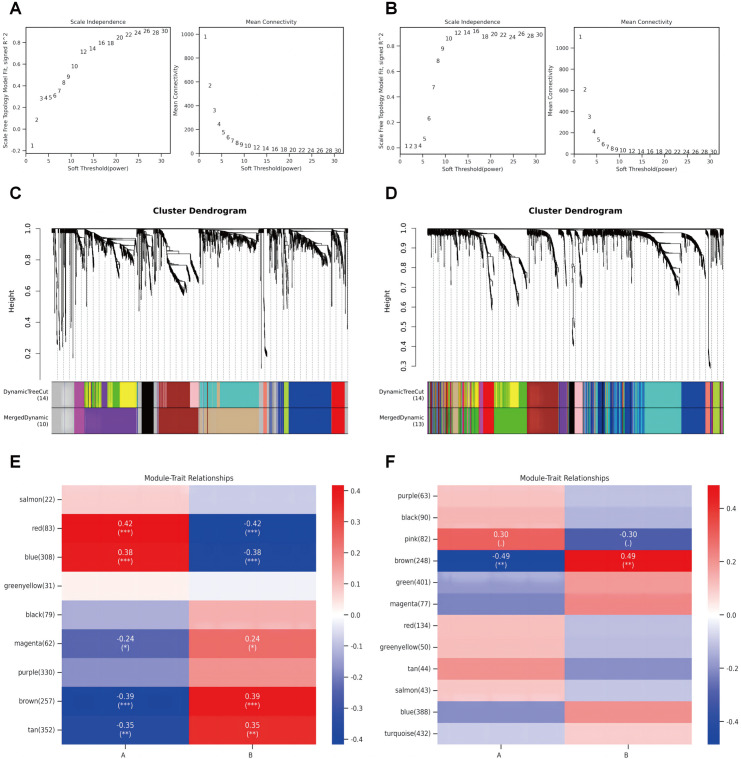
WGCNA and key module identification in AS and uveitis. **(A)** Soft threshold power determination for the AS cohort (*β* = 20). **(B)** Soft threshold power determination for the uveitis cohort (*β* = 10). **(C, D)** Cluster dendrograms of highly connected genes in AS and uveitis key modules. **(E, F)** Module-trait correlation heatmaps for AS and uveitis, highlighting disease-associated modules.

We employed weighted gene co-expression network analysis (WGCNA) to delineate disease-associated gene clusters and quantify their correlation with clinical phenotypes. For the AS cohort, a soft thresholding power of *β* = 20 was selected to ensure scale-free topology (scale-free **R*^*2*^ *> 0.83; Fig 4A), while the uveitis cohort required *β *= 10 for optimal network construction (Fig 4B). Following dynamic tree cutting and module merging, 9 co-expression modules were identified in AS and 12 in uveitis. Module-trait analysis revealed that the brown module (correlation coefficient *r* = 0.39, **p <* *0.001) and tan module (*r* = 0.35, **p <* *0.01) exhibited the strongest positive associations with AS diagnosis (Fig 4C, 4E). In the uveitis cohort, the brown module demonstrated the highest correlation with disease status (**r* *= 0.49, **p <* *0.01; Fig 4D, 4F).

### Identification of shared hub genes via machine learning algorithms

**Fig 5:**
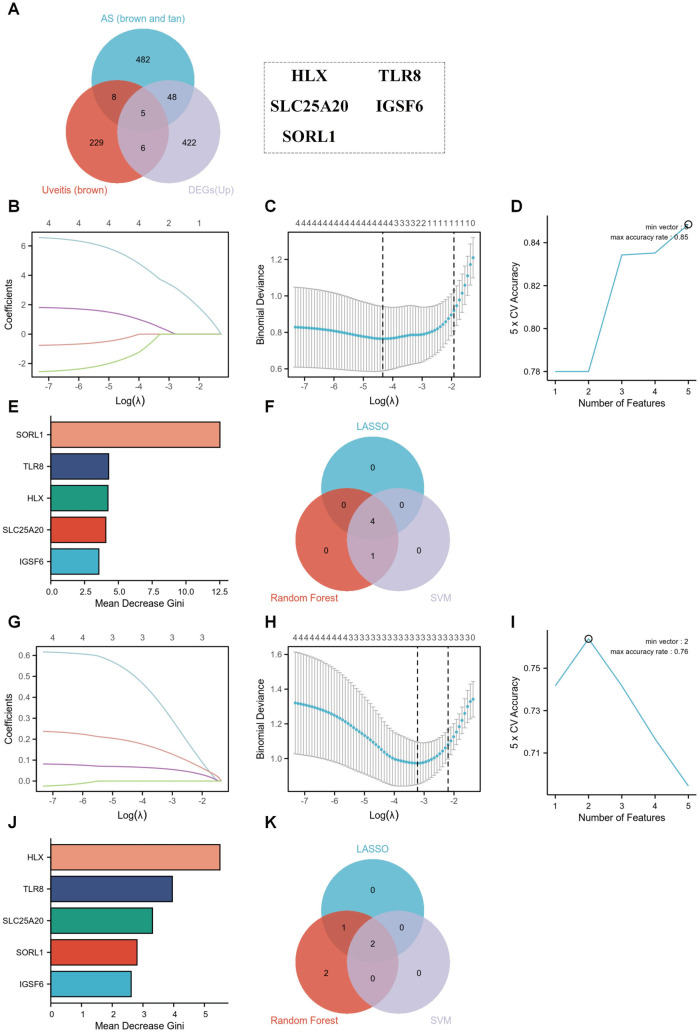
Machine learning-driven identification of shared hub genes. **(A)** Venn diagram of 5 overlapping genes from DEGs and WGCNA modules. **(B, C)** LASSO coefficient profiles and optimal biomarker selection for AS. **(D)** SVM-RFE accuracy curve identifying 5 diagnostic genes for AS. **(E)** Random forest error rates and variable importance ranking for AS. **(F)** Intersection of LASSO, SVM-RFE, and RF-selected AS biomarkers. **(G, H)** LASSO coefficient profiles and biomarker selection for uveitis **(I)** SVM-RFE accuracy curve identifying 2 diagnostic genes for uveitis. **(J)** Random forest error rates and variable importance ranking for uveitis. **(K)** Consensus biomarkers (HLX/SLC25A20) from multi-algorithm intersection.

To identify core regulatory genes shared between AS and uveitis, we first intersected genes from WGCNA-derived disease-associated modules and cross-disease DEGs, yielding five candidate genes: SORL1, TLR8, HLX, SLC25A20, and IGSF6 (Fig 5A). These candidates were subsequently refined using three machine learning algorithms. As shown in Fig 5B, 5C, machine learning refinement in the AS cohort revealed four biomarkers (SORL1, TLR8, HLX, SLC25A20) through LASSO regression, while SVM-RFE retained all five genes with 85% accuracy (Fig 5D). Random Forest prioritized SORL1 (Fig 5E), with intersection confirming these four genes (Fig 5F). Parallel analysis in uveitis identified HLX and SLC25A20 as consensus biomarkers across LASSO (Fig 5G, 5H), SVM-RFE (Fig 5I), and Random Forest (Fig 5J), validated through multi-algorithm intersection (Fig 5K). These results position HLX and SLC25A20 as robust transcriptional regulators bridging AS-uveitis comorbidity.

### Validation of core biomarkers HLX and SLC25A20

**Fig 6:**
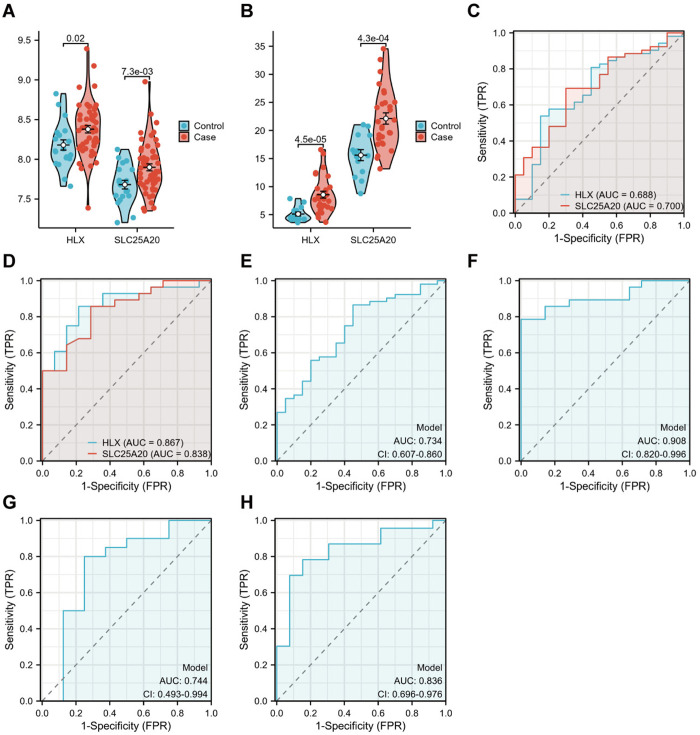
Validation of core biomarkers HLX and SLC25A20. **(A, B)** Differential expression of HLX and SLC25A20 in AS (GSE73754) and uveitis (GSE194060). **(C, D)** ROC curves of HLX and SLC25A20 in AS (AUC = 0.688/0.700) and uveitis (AUC = 0.867/0.838). **(E, F)** Generalized linear model ROC analysis for combined biomarkers in AS (GSE73754) and uveitis (GSE194060). **(G, H)** External validation ROC curves in AS (GSE221786) and uveitis (GSE195501).

Intersection of machine learning results across AS and uveitis cohorts identified HLX and SLC25A20 as core diagnostic biomarkers, with both genes demonstrating significant upregulation in disease groups ( *p <* 0.05; Fig 6A, 6B). ROC analysis revealed robust discriminatory capacity for AS diagnosis (HLX-AUC = 0.688; SLC25A20-AUC = 0.700) and superior performance in uveitis (HLX-AUC = 0.867; SLC25A20-AUC = 0.838; Fig 6C, 6D), and a generalized linear model integrating both biomarkers further confirmed their reliability (AS-AUC = 0.734; uveitis-AUC = 0.908; Fig 6E, 6F). External validation using independent cohorts (GSE221786 for AS: AUC = 0.744; GSE195501 for uveitis: AUC = 0.836; Fig 6G, 6H). These findings underscore HLX and SLC25A20 as high-confidence transcriptional discriminators of AS-uveitis comorbidity.

### Immune landscape profiling in AS and uveitis

**Fig 7:**
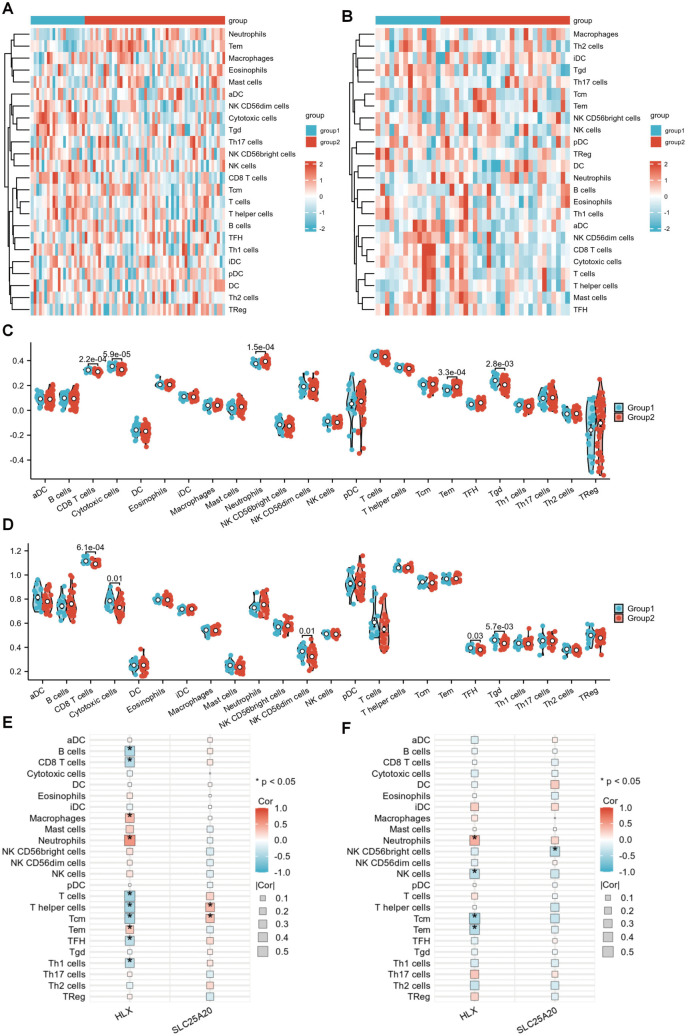
Immune landscape profiling in AS and uveitis. **(A, B)** Heatmaps showing immune cell abundance differences between healthy controls and AS/uveitis patients. **(C, D)** Violin plots comparing 24 immune cell subsets in AS and uveitis versus controls. **(E, F)** Spearman correlation matrices between hub genes (HLX/SLC25A20) and immune cells in AS **(E)** and uveitis **(F)**. * **p <* *0.05.

Given the pivotal role of immune dysregulation in both uveitis and AS, we employed the ssGSEA algorithm to quantify immune infiltration patterns. Comparative analysis revealed elevated neutrophils (*p <* 0.01) and effector memory T (Tem) cells *(p <* 0.01) infiltration in AS patients versus controls (Fig 7A, 7C). Conversely, uveitis tissues exhibited distinct immune profiles characterized by reduced NK CD56dim cells and T follicular helper (TFH) subsets (*p <* 0.05; Fig 7B, 7D). Notably, CD8^+^ T cells, Cytotoxic cells and γδ T (Tgd) subsets depletion emerged as convergent immunological hallmarks across both diseases.

### Core biomarker-immune interactions

Spearman correlation analysis revealed stark contrasts in biomarker-immune interactions. In AS cohort, HLX positively correlated with neutrophils (*r* = 0.55, *p* *<* 0.001) but inversely with T cells (*r* = −0.52, *p* *<* 0.001), while SLC25A20 associated with T helper cells (*r* = 0.36, *p* *<* 0.01) and central memory T (Tcm) cells (*r* = 0.32, *p* *<* 0.05; Fig 7E). In Uveitis cohort, HLX maintained neutrophil correlation (**r* *= 0.41, **p <* *0.05) yet inversely linked to Tcm cells (*r* = −0.50, *p <* 0.01). SLC25A20 showed significant linkage to NK CD56bright cells (*r* = −0.40, **p <* *0.05; Fig 7F). These results position HLX and SLC25A20 as immunologic regulators, balancing pro-inflammatory neutrophil recruitment and lymphoid cell homeostasis in AS-uveitis comorbidity.

### Chemical screening and molecular docking targeting HLX and SLC25A20 expression

**Fig 8:**
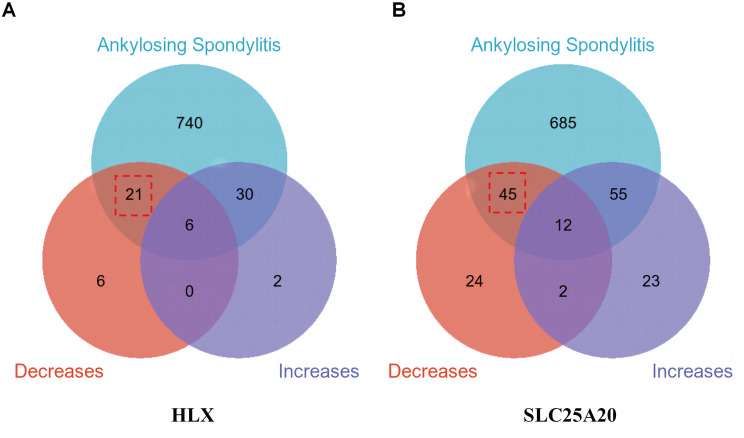
Screening for chemicals association with HLX and SLC25A20. **(A)** Venn diagram of the chemicals associated with HLX expression in AS. **(B)** Venn diagram of the chemicals associated with SLC25A20 expression in AS.

To explore HLX and SLC25A20 as potential therapeutic targets for AS, we gathered chemical modulators of their expression from the Comparative Toxicogenomics Database (CTD; https://ctdbase.org/), along with compounds associated with AS (S1–S3 Tables). Our dataset comprised 33 chemicals that downregulate HLX and 38 that upregulate it (Fig 8A), as well as 83 agents that downregulate SLC25A20 and 92 that upregulate it (Fig 8B). By performing intersection analysis with AS-related compounds, we identified 21 chemicals that suppress HLX (Fig 8A) and 45 candidates that suppress SLC25A20 (Fig 8B).

**Fig 9:**
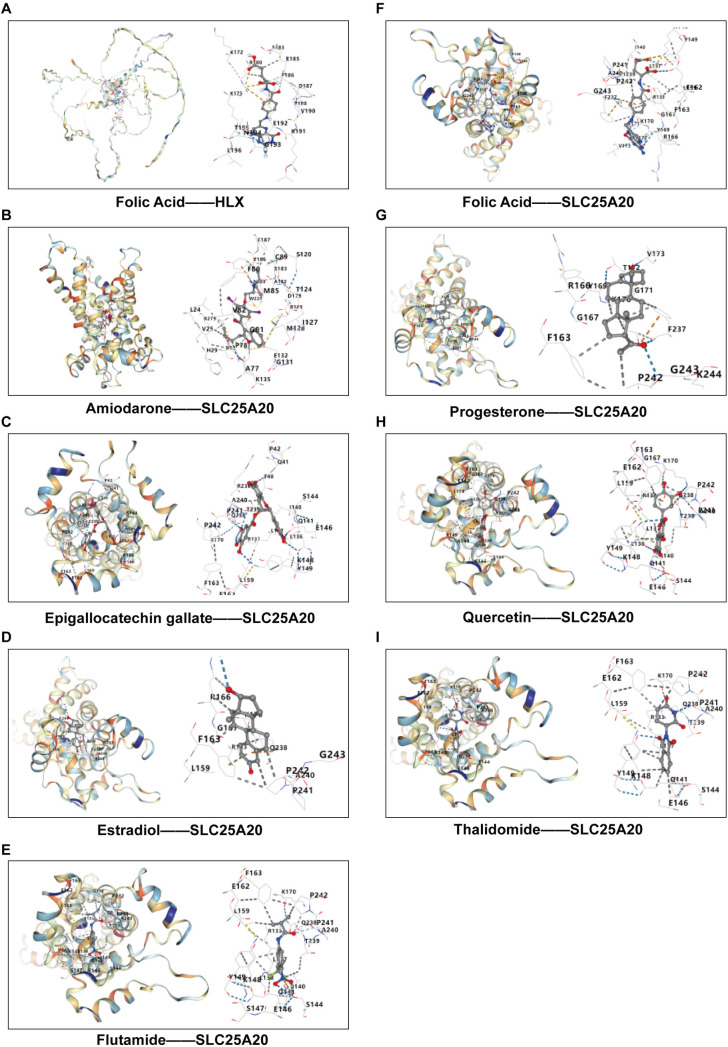
Molecular docking results for each drug with HLX and SLC25A20. **(A)** Folic Acid and HLX, **(B)** Amiodarone and SLC25A20, **(C)** Epigallocatechin gallate and SLC25A20, **(D)** Estradiol and SLC25A20, **(E)** Flutamide and SLC25A20, **(F)** Folic Acid and SLC25A20, **(G)** Progesterone and SLC25A20, **(H)** Quercetin and SLC25A20, and **(I)** Thalidomide and SLC25A20.

Because HLX and SLC25A20 are highly expressed in AS, indicating that reducing HLX and SLC25A20 expression may be a possible therapeutic strategy for AS. Among the 21 HLX inhibitors, 6 were found to be clinically approved drugs (S4 Table). Likewise, we identified 13 clinically approved therapeutics among the 45 SLC25A20 inhibitors (S4 Table). Molecular docking studies revealed that Folic Acid achieved a Vina score of −7.5 kcal/mol with HLX (Fig 9A, S4 Table), while Amiodarone, Epigallocatechin gallate, Estradiol, Flutamide, Folic Acid, Progesterone, Quercetin, and Thalidomide recorded Vina scores of −8.0, −8.5, −7.7, −7.0, −8.2, −7.3, −7.0, and −7.1 kcal/mol, respectively, with SLC25A20 (Fig 9B–9I, S4 Table). These findings highlight the therapeutic potential of these compounds in the intervention of AS.

## Discussion

Ankylosing Spondylitis (AS) is a chronic inflammatory disease primarily affecting the axial skeleton, leading to significant morbidity [1,2]. It is characterized by the presence of sacroiliitis and can progress to fusion of the spine, resulting in a loss of mobility and quality of life. Up to 40% of AS patients experience extra-articular manifestations, with uveitis being one of the most common, leading to severe ocular complications if left untreated [3,4]. The relationship between AS and uveitis is complex, as both conditions share a strong association with HLA-B27 positivity, yet the specificity and sensitivity of HLA-B27 in diagnosing uveitis in AS patients remain low [5–7]. Understanding the underlying molecular and immunological mechanisms linking these two diseases is crucial for improving diagnostic and therapeutic approaches.

This study aims to identify cross-disease biomarkers and pathways between AS and uveitis through a comprehensive analysis of peripheral blood transcriptomic data. By employing differential expression analysis, co-expression networks, and machine learning algorithms, we identified shared dysregulated genes and their potential functional significance. Notably, we found 481 upregulated and 216 downregulated genes common to both diseases, with HLX and SLC25A20 emerging as core biomarkers. This research not only elucidates the shared molecular pathways between AS and uveitis but also highlights the potential of these biomarkers for early diagnosis and targeted therapeutic strategies.

The identification of shared dysregulated genes between AS and Uveitis provides essential insights into the molecular mechanisms underlying these interconnected diseases. Our analysis revealed 481 upregulated and 216 downregulated DEGs in the peripheral blood of patients with AS and Uveitis. The upregulated genes were primarily enriched in biological processes such as the regulation of GTPase activity and protein polyubiquitination, while the downregulated genes were associated with ribosome biogenesis and oxidative phosphorylation. These findings align with previous research that highlights the importance of inflammatory pathways in both diseases, suggesting a shared dysregulation of immune responses that could contribute to disease pathogenesis [11–17]. The overlap of these DEGs not only supports the notion of a common pathway but also emphasizes the potential for identifying biomarkers that could aid in the diagnosis and treatment of patients suffering from these co-occurring conditions.

In addition to identifying DEGs, our functional enrichment analysis underscored the relevance of specific biological pathways implicated in the pathogenesis of both AS and Uveitis. The significant enrichment of genes involved in the Toll-like receptor signaling pathway and immune response regulation indicates that dysregulation within these pathways may play a pivotal role in the development and exacerbation of both conditions [17–19]. These pathways are known to be central to the inflammatory responses observed in AS and Uveitis, suggesting that targeting these pathways could provide therapeutic avenues for managing both diseases. Furthermore, the shared involvement of oxidative phosphorylation in the downregulated genes may suggest a metabolic alteration common to both diseases, highlighting the need for further investigation into the metabolic implications of AS and uveitis.

The establishment of co-expression networks through WGCNA revealed significant modules of co-expressed genes that correlate with disease characteristics. The identification of specific modules positively associated with AS and Uveitis reinforces the idea that there are distinct gene interactions that could be pivotal in understanding the interplay between these diseases. Moreover, the positive and negative correlations observed in disease-specific modules suggest that certain clusters of genes may contribute to the pathogenesis of AS or uveitis in different ways, warranting further exploration of these modules as potential therapeutic targets or biomarkers. Overall, our findings emphasize the complexity of the molecular interactions between AS and Uveitis and the need for a deeper understanding of these relationships to inform future research and clinical practice.

The findings from our study highlight the critical role of HLX and SLC25A20 as potential cross-disease biomarkers that bridge AS and uveitis. The elevation of these biomarkers in the peripheral blood of patients suffering from both conditions suggests a shared pathogenic mechanism that warrants further exploration. The association of HLX with Th17 cell differentiation indicates a possible link to immune dysregulation [20,21], which is a hallmark of both AS and uveitis. Furthermore, SLC25A20, known for its involvement in mitochondrial function and fatty acid transport [22–24], may contribute to the metabolic changes observed in AS patients, signaling a potential avenue for therapeutic intervention. Additionally, molecular docking studies revealed that folic acid acts as a strong suppressor of HLX, with a Vina score of −7.5 kcal/mol, while amiodarone, epigallocatechin gallate, estradiol, flutamide, folic acid, progesterone, quercetin, and thalidomide were identified as suppressor of SLC25A20, with scores of −8.0, −8.5, −7.7, −7.0, −8.2, −7.3, −7.0, and −7.1 kcal/mol, respectively. This suggests that these compounds could be repurposed as therapeutic options for AS intervention. Although our findings position HLX/SLC25A20 as promising therapeutic targets, preclinical validation of their disease-modifying mechanisms remains essential for clinical translation.

This study has several limitations. First, the functional roles of HLX and SLC25A20 remain to be experimentally validated through wet-lab approaches, as bioinformatic evidence alone cannot fully confirm biological relevance. Second, our dependence on GEO datasets rather than prospectively collected cohorts may introduce biases in age stratification, sex distribution, disease staging heterogeneity, and ancestry representation. Third, the bulk transcriptome-based immune infiltration analysis lacks single-cell resolution; future investigations using PBMC scRNA-seq datasets could more precisely delineate immune cell dynamics. Future studies should address these constraints through functional validation of biomarkers, multi-center cohorts with balanced demographic sampling, and single-cell resolution analyses to advance clinical translation.

In conclusion, this study successfully identifies HLX and SLC25A20 as core biomarkers linking AS and uveitis. The findings underscore their potential role in the shared pathophysiological mechanisms of these diseases, offering a promising avenue for early diagnosis and targeted therapeutic strategies. By elucidating the functional implications of these biomarkers and their relationship with immune response, this research lays the groundwork for future investigations aimed at improving clinical outcomes for patients suffering from these interconnected inflammatory conditions.

## Methods

### Data acquisition and differential expression analysis

The datasets GSE73754 and GSE194060 were retrieved from the Gene Expression Omnibus (GEO; https://www.ncbi.nlm.nih.gov/geo/). GSE73754 comprised 52 peripheral blood samples from ankylosing spondylitis (AS) patients and 20 healthy controls, generated using the Illumina HumanHT-12 V4.0 platform (GPL10558). GSE194060 included 28 peripheral blood mononuclear cell (PBMC) samples from uveitis patients and 14 controls, sequenced on the Illumina NovaSeq 6000 platform (GPL24676). The platform and grouping information about the datasets brought into this study was exhibited in S5 Table. Differentially expressed genes (DEGs) were identified using the limma R package (v3.52.2) with a significance threshold of *p <* 0.05.

### Functional enrichment profiling

Gene Ontology (GO) and Kyoto Encyclopedia of Genes and Genomes (KEGG) analyses were performed to annotate DEG-associated biological functions. GO terms were categorized into Molecular Functions (MF), Biological Processes (BP), and Cellular Components (CC). Enriched pathways were defined by adjusted *p <* 0.05.

### Weighted gene co-expression network analysis (WGCNA)

WGCNA was implemented to identify disease-associated gene modules. Genes within the top 35% of variance were retained, and outlier samples were excluded via hierarchical clustering. A soft threshold power (*β *= 20 for AS, *β* = 10 for uveitis) was selected to achieve scale-free topology (**R*^*2*^* >** 0.80). Dynamic tree cutting with a minimum module size of 20 and merge threshold of 0.3 generated distinct co-expression modules. The topological overlap matrix (TOM) was preserved for network visualization. Module-trait correlations were quantified by calculating module eigengenes and their Pearson correlation coefficients with clinical phenotypes (*p <* 0.05).

### Machine learning algorithms

Three machine learning approaches were implemented to prioritize diagnostic biomarkers:

**LASSO Regression:** The glmnet package (v4.1.7) performed L1-penalized regression with 10-fold cross-validation (cv.glmnet) to optimize λ (range: 0–100). The binomial family and α = 1 (pure LASSO) were specified.
**SVM-RFE:** Using the e1071 package (v1.7.13), linear kernel support vector machines with cost parameters (1–20) were tuned via grid search. Feature ranking was determined by recursive feature elimination.
**Random Forest:** The randomForest package (v4.7.1.1) generated 500 decision trees (mtry = √n_features) with minimum error rate optimization via tuneRF (ntree range: 0–700, step = 1).

### Diagnostic model construction

Logistic regression analysis evaluated associations between candidate biomarkers (predictors) and clinical status (binary outcome: patients vs. healthy controls). Multivariate logistic regression was implemented via the glm function in R (v4.2.1) to assess the diagnostic utility of biomarker combinations. Model performance was quantified using receiver operating characteristic (ROC) curves, with area under the curve (AUC) > 0.7 considered clinically informative.

### Immune microenvironment profiling

Single-sample gene set enrichment analysis (ssGSEA) was conducted using the GSVA package (v1.48.0) with 24 immune cell signatures curated from published compendia [25,26]. Spearman correlations between hub gene expression and immune cell abundance were calculated (*p <* 0.05).

### Chemical screening and molecular docking

Candidate compounds modulating HLX and SLC25A20 expression, along with therapeutics associated with ankylosing spondylitis (AS), were curated from the Comparative Toxicogenomics Database (CTD; https://ctdbase.org/). Three-dimensional structures of HLX (UniProt: Q14774) and SLC25A20 (UniProt: O43772) were retrieved from the AlphaFold Protein Structure Database (https://alphafold.ebi.ac.uk/) for receptor preparation. Ligand structures were acquired from PubChem (https://pubchem.ncbi.nlm.nih.gov/) and energy-minimized using molecular mechanics force fields. Molecular docking was performed on the CB-Dock2 platform (https://cadd.labshare.cn/cb-dock2/), which integrates curvature-based cavity detection and template-guided docking algorithms. Binding poses were prioritized based on Vina scores (≤−7 kcal/mol indicating strong ligand-receptor interactions) and ensuring they matched or slightly exceeded the dimensions of the ligands.

### Statistics

All analyses were executed in R 4.2.1. Multiple testing correction used Benjamini-Hochberg FDR, with significance defined as *p <* 0.05 or FDR* *<** 0.1 where applicable.

## Supporting information

S1 Table The list of chemicals affecting HLX expression from the CTD database. (XLSX)

S2 Table The list of chemicals affecting SLC25A20 expression from the CTD database. (XLSX)

S3 Table The list of chemicals related to ankylosing spondylitis was downloaded from the CTD database. (XLSX)

S4 Table Molecular docking details of chemicals in association with HLX and SLC25A20. (XLSX)

S5 Table Detailed information on ankylosing spondylitis and uveitis databases. (XLSX)
